# Multi-omics analysis of the metabolic and transcriptional regulatory mechanisms underlying strontium-induced anthocyanin accumulation in fresh purple maize

**DOI:** 10.3389/fpls.2026.1777794

**Published:** 2026-03-12

**Authors:** Zhao Binhan, Pu Rumin, Mei Yuqin, Rui Lin, Deng Hanyu, Zhou Wenhao, Xiang Wenqi, Liu Mao, Wei Chengzhi, Lu Yanli, Wang Qingjun, Li Jingwei, Rui Tijiang, Li Jizhang, Lin Haijian

**Affiliations:** 1 Sichuan Agricultural University, Chengdu, Sichuan, China; 2 Lincang Seed Management Station, Lincang, Yunnan, China; 3 Shandong Agricultural University, Taian, Shandong, China; 4 Lijiang Seed Management Station, Lijiang, Yunnan, China

**Keywords:** anthocyanins, fresh purple maize, metabolomics, strontium, transcriptomics

## Abstract

Fresh purple corn (*Zea mays* L.) is rich in anthocyanins, and enhancing its anthocyanin content is crucial for improving its nutritional profile and commercial appeal. Strontium (Sr), an essential human micronutrient involved in physiological processes such as bone formation, exhibits low dietary bioavailability, making the consumption of Sr-enriched agricultural products a critical route for Sr supplementation. Given the biphasic effect of strontium on plant growth, characterized by low-dose stimulation and high-dose inhibition, elucidating its regulatory mechanism in fresh purple corn anthocyanin biosynthesis and breeding cultivars with dual high strontium and anthocyanin traits represents a biofortification strategy with considerable application potential. In this study, a pot experiment was conducted with fresh purple corn, and the anthocyanin and strontium contents in the kernels were quantified. To characterize the dynamic expression profiles of anthocyanin-related genes and metabolite accumulation under strontium treatment, an integrated transcriptomic and metabolomic approach was employed. Key differentially expressed genes were further validated by qRT-PCR. The results demonstrated that strontium treatment significantly increased the anthocyanin content in purple corn kernels relative to the control. Integrated multi-omics analysis revealed that strontium promotes anthocyanin synthesis by activating genes such as *Zm00001d003015*, *Zm00001d015513* and *Zm00001d016471*, enhances the accumulation of pivotal hub metabolites (such as naringin chalcone, 4-coumaric acid), and optimizes the allocation of glycosylation donors by suppressing competing metabolic pathways such as coumarin biosynthesis. Collectively, this study provides the first systematic elucidation of how strontium, as a novel inducer, promotes anthocyanin synthesis in purple corn through a multi-level, temporally regulated network, providing a theoretical foundation and a practical biofortification strategy for developing Sr-enriched, high-anthocyanin maize.

## 1 Introduction

In the intricate interplay between plants and human health, strontium (Sr) and anthocyanins represent two critical components: the former an essential trace element vital for physiological functions (Ru et al., 2024), and the latter a plant secondary metabolite endowed with remarkable health-promoting properties (Gao et al., 2025). Due to its chemical similarity to calcium, strontium plays an irreplaceable role in maintaining bone mineral density and supporting neural conductivity (Borciani et al., 2022). However, dietary sources of strontium are limited, and its low bioavailability often leads to insufficient daily intake. Meanwhile, anthocyanins, a class of water-soluble flavonoids widely distributed in plants, exert substantial health benefits, particularly in the prevention of cardiovascular diseases, metabolic syndrome, and neurodegenerative disorders, attributable to their potent antioxidant, anti-inflammatory, and cytoprotective activities (Li et al., 2024; Cappellini et al., 2021). Freshly consumed purple corn, as a natural specialty crop abundant in anthocyanins, is not only visually appealing but also considered an ideal raw material for functional food development (Lao et al., 2017).

Notably, these two substances may share intrinsic physiological connections within plants beyond their independent roles. Preliminary studies suggest that certain trace elements, at appropriate concentrations, can act as signaling molecules to activate or enhance specific secondary metabolic pathways in plants (Chao et al., 2024). For instance, in *Amaranthus tricolor*, strontium application has been shown to induce anthocyanin accumulation (Cheng et al., 2022). This observation leads to a scientifically compelling and application-oriented hypothesis: exogenous strontium might serve both as a nutritional element for enriching corn kernels and as an effective physiological signal to directionally upregulate the anthocyanin biosynthesis pathway. Such a dual role may achieve synergistic outcomes of “nutrient fortification” and “functional enhancement” (Hong et al., 2023). If validated, this approach would enable a single agronomic intervention to simultaneously improve two high-value attributes of fresh corn, thereby offering a novel strategy for developing compound nutrient-enhanced functional products.

Nevertheless, current understanding of this potential synergistic mechanism remains limited. Research on strontium in plants has historically centered on its environmental behavior (Sasmaz et al., 2020), toxicity thresholds (Zhang et al., 2020), or its role as a calcium analogue in basic physiological processes (Sahin et al., 2024). However, a systematic, time-resolved molecular-level understanding of how strontium precisely regulates complex secondary metabolic networks, such as the phenylpropanoid-flavonoid pathways, and specifically the dynamic synthesis of its key product anthocyanins, is still lacking. This knowledge gap severely constrains our ability to harness strontium for the targeted improvement of crop nutritional quality.

To address this, the present study directly investigates the core “strontium-anthocyanins” relationship using fresh purple corn as a model system, with the aim of elucidating the intrinsic molecular logic underlying strontium-induced anthocyanin synthesis. We integrated dynamic physiological assessments with transcriptomic and metabolomic analyses to capture the full-chain response across multiple temporal stages, from strontium absorption and signal transduction to the activation of anthocyanin synthesis pathways. This study not only seeks to uncover the specific regulatory networks through which strontium acts as a novel inducer at both transcriptional and metabolic levels, but also aims to clarify how it optimizes anthocyanin accumulation by reshaping resource allocation and coordinating competing metabolic fluxes. The findings will provide a solid theoretical foundation and key molecular targets for designing and cultivating “strontium-anthocyanin” synergistic-enhanced maize varieties, thereby contributing novel strategies for nutrition-oriented precision agriculture.

## 2 Materials and methods

### 2.1 Experimental materials and treatment

The purple corn inbred line SSD6047 (Crystal Black Sweet) was used as the experimental material. The exogenous treatment experiment was designed with reference to and modifications based on the method described by Chang et al (Chang et al., 2017). Two treatment groups were established: the control group (CK, without strontium addition) and the strontium treatment group (Sr, with Sr added at 250 mg/kg). strontium chloride (SrCl_2_≥6H_2_O, ≥99.9% purity) was dissolved in purified water and evenly applied around the corn roots two days after pollination. Each treatment consisted of three biological replicates, with a cultivation period of 27 days. Grain samples were collected at 15, 17, 19, 21, 23, 25, and 27 days after pollination, then immediately frozen in liquid nitrogen, and stored at -80 °C for subsequent analysis.

### 2.2 Quantification of strontium content

Strontium ion content was measured using Inductively Coupled Plasma Mass Spectrometry (ICP-MS) with minor modifications to the detection procedure. Briefly, fresh maize kernel samples were accurately weighed and digested in a microwave digester. Sr content was then quantified using the ICP-MS instrument. Quantitative analysis was performed via the standard curve method, with each sample measured in triplicate to ensure data accuracy.

### 2.3 Assay of total anthocyanin content

Total anthocyanin content was determined based on the pH differential method with slight modifications. Briefly, 0.5 g of frozen kernel samples was accurately weighed, and 10 mL of extraction solution (80% methanol containing 0.1% HCl) was added. Ultrasound-assisted extraction was conducted for 30 minutes, followed by centrifugation at 4 °C and 8000 rpm for 10 minutes to collect the supernatant. The supernatant was diluted with pH 1.0 KCl-HCl buffer and pH 4.5 NaAc-HAc buffer, respectively. Absorbance was measured at 520 nm, and total anthocyanin content was calculated using the respective formula. Each sample was analyzed with three technical replicates.

### 2.4 Transcriptome analysis

Maize kernel samples from seven time points were selected for total RNA extraction and mRNA enrichment. mRNA fragments were synthesized into cDNA. During second-strand synthesis, dUTP was incorporated for strand labeling, followed by digestion with UDG enzyme to construct a strand-specific cDNA library. The library was circularized and replicated via rolling circle amplification to form DNA Nanoballs (DNBs), followed by high-throughput sequencing. Strict quality control was performed on the raw reads. Valid reads passing quality control were aligned to the reference genome. Principal component analysis (PCA), correlation analysis, and differentially expressed genes (DEGs) identification were conducted based on gene expression levels. DEGs were subjected to Gene Ontology (GO) functional enrichment analysis, Kyoto Encyclopedia of Genes and Genomes (KEGG) pathway enrichment analysis, clustering analysis, and transcription factor prediction (Cock et al., 2009).

### 2.5 Metabolome analysis

Metabolomic profiling was conducted using a liquid chromatography-tandem mass spectrometry (LC-MS) platform. For sample preparation, fresh corn kernel samples collected at the seven time points were accurately weighed and transferred into centrifuge tubes. An 800 μL aliquot of pre-chilled extraction solvent (methanol:water = 7:3, v/v) and 20 μL of internal standard solution (containing d3-Leucine, etc.) were added. The mixture was homogenized in a ball mill at 50 Hz for 10 min, followed by ultrasonic-assisted extraction for 30 min in an ice-water bath at 4 °C. After incubation at -20 °C for 1 h, the extract was centrifuged. The supernatant was filtered through a 0.22 μm microporous membrane, and a portion of the filtrate was pooled to prepare quality control samples. High-resolution mass spectrometry (QExactive) coupled with a liquid chromatography system was used to collect primary and secondary mass spectrometry data in both positive and negative ion modes. The raw data were processed using Compound Discoverer software for peak extraction, alignment, and normalization. Metabolite identification and annotation were conducted by integrating with the BMDB, mzCloud, and ChemSpider databases.

### 2.6 qRT-PCR validation

Quantitative real-time fluorescent PCR (qRT-PCR) was conducted to validate the key differentially expressed genes identified from screening, RNA extraction using corn kernels. Primers were designed based on the transcript sequences of the target genes using the NCBI Primer-BLAST (**Supplementary Table 2**) and synthesized by Shanghai Bioengineering Co., Ltd. The GAPDH and β-Actin genes were selected as internal references, as both have been widely documented to show stable expression across different maize tissues and developmental stages, particularly during grain development, making them suitable for expression standardization in this study.

Genomic DNA was removed and the first-strand cDNA was synthesized using the PrimeScript™ RT Reagent Kit with gDNA Eraser (TaKaRa). The qRT-PCR reaction was performed on a QuantStudio 3 Real-Time PCR System with SYBR^®^ Premix Ex Taq™ II (Tli RNaseH Plus). Each 20 μL reaction consisted of 10 μL SYBR Premix Ex Taq II, 0.8 μL each of forward and reverse primers (10 μM each), 2 μL of cDNA template (diluted 50-fold), and 6.4 μL RNase-free water. The thermal cycling conditions were as follows: 95 °C pre-denaturation for 2 min, followed by 40 cycles of 95 °C for 15 s, 56 °C for 30 s, and 72 °C for 30 s. All reactions were run with at least three replicates per sample. Relative expression levels were calculated using the 2−ΔΔCT method.

### 2.7 Statistical analysis

All experimental data were organized using Excel 2021. Statistical analysis and data visualization were conducted with GraphPad Prism 9.0. Inter-group comparisons were assessed via independent samples t-tests or two-way analysis of variance (ANOVA). Correlation analysis was performed using the Spearman rank correlation method, with *P* < 0.05 considered statistically significant.

## 3 Results

### 3.1 Determination of anthocyanin and strontium content and phenotype of grains

**Figure 1:**
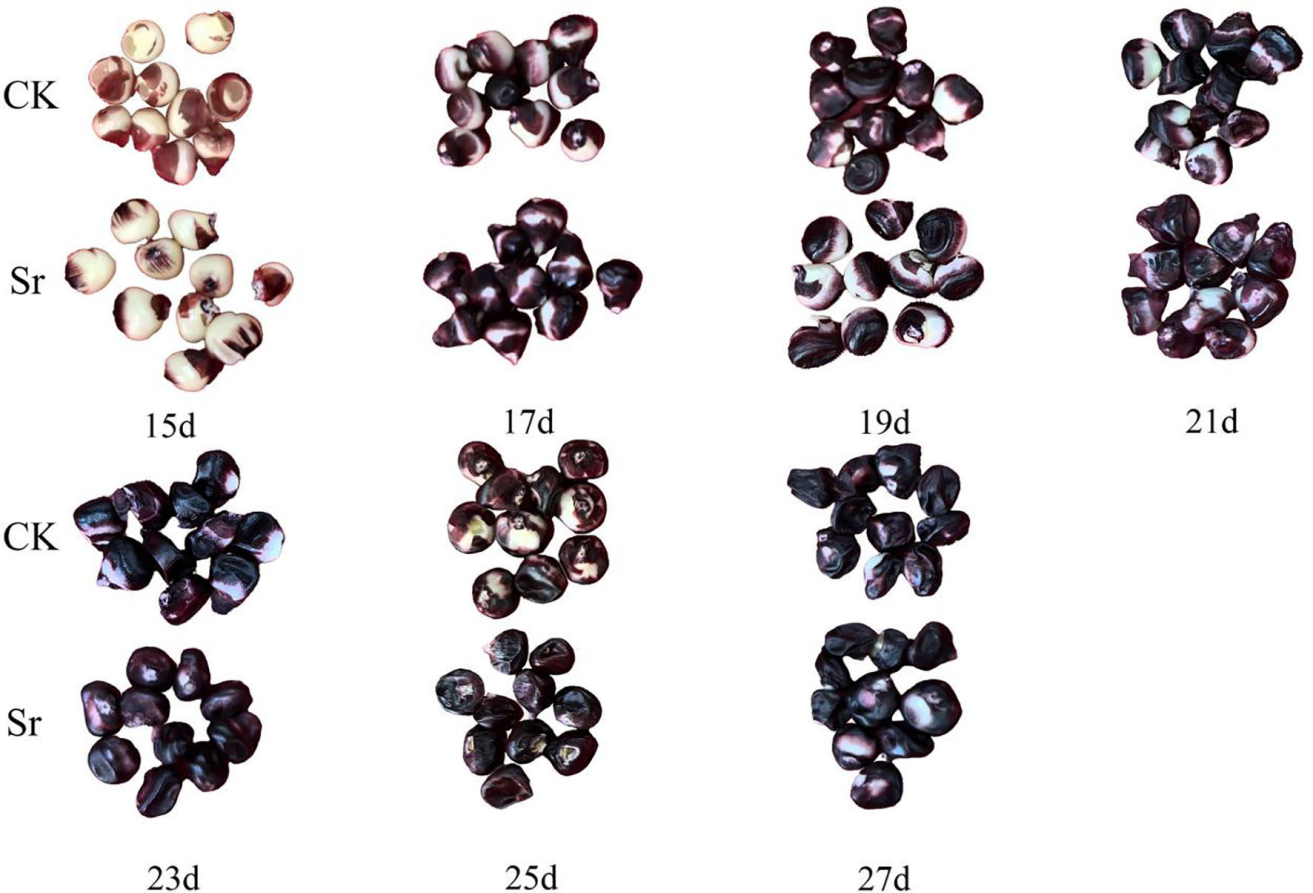
Comparison of corn seeds.

Strontium (Sr) treatment led to a shift in the visual phenotype of fresh purple corn kernels (**Figure 1**). Compared with the control (CK), the intensified purple hue in Sr-treated kernels points toward a higher concentration of anthocyanins.

**Figure 2:**
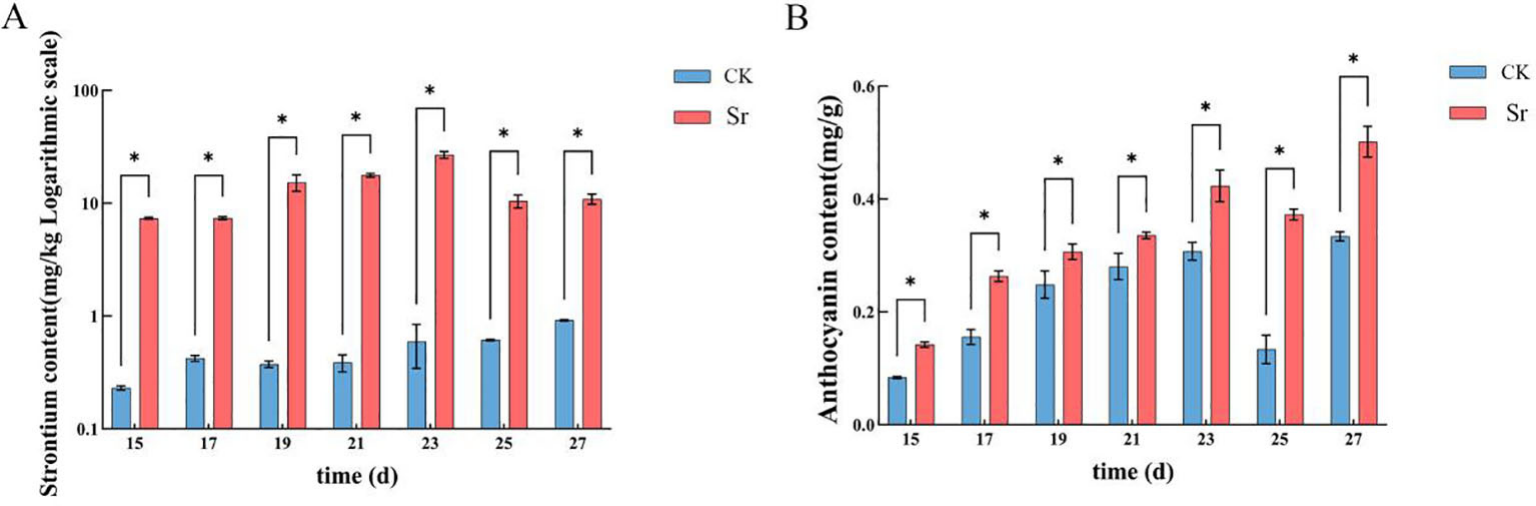
**(A)** illustrates strontium content in maize kernels (mg/kg), where CK denotes the control group and CK the strontium-treated group. **(B)** displays anthocyanin levels, where the vertical axis indicates anthocyanin concentration (mg/g), with CK representing the control group and CK representing the treatment group. Data are presented as mean ± standard deviation (n = 3). Differences between CK and CK at each time point were assessed using an independent samples t-test (*p < 0.05, **p < 0.01).

Measurement of Sr and anthocyanin content at seven time points during kernel development revealed that both were consistently and significantly higher in the Sr treatment group (Sr) than those in the control group (CK) throughout the experimental period. As depicted in **Figure 1**, the Sr content in the Sr group showed a gradual upward trend with development, reaching a peak at 21 DAP (31.0247 mg/kg) and remaining relatively stable thereafter (**Figure 2A**). Anthocyanin content exhibited distinct stage-wise accumulation characteristics: 15–17 DAP was the initial accumulation phase, 19–21 DAP was the rapid accumulation phase, and the peak was reached at 23–27 DAP (**Figure 2B**). Notably, the peak of Sr accumulation preceded that of anthocyanin synthesis. This temporal sequence indicates that Sr likely functions as an upstream signal initiating the anthocyanin biosynthetic cascade (Ramadani and Jadid, 2024).

### 3.2 Screening of differential metabolites and correlation analysis with anthocyanin synthesis

Following metabolome data preprocessing, a total of 16,882 metabolites were identified. Among these, 80% of the metabolites exhibited a coefficient of variation (CV) ≤ 30%, and the correlation coefficient (R) between quality control (QC) samples was ≥ 0.993, indicating good reproducibility of the experimental data that met the criteria for subsequent analyses.

**Figure 3:**
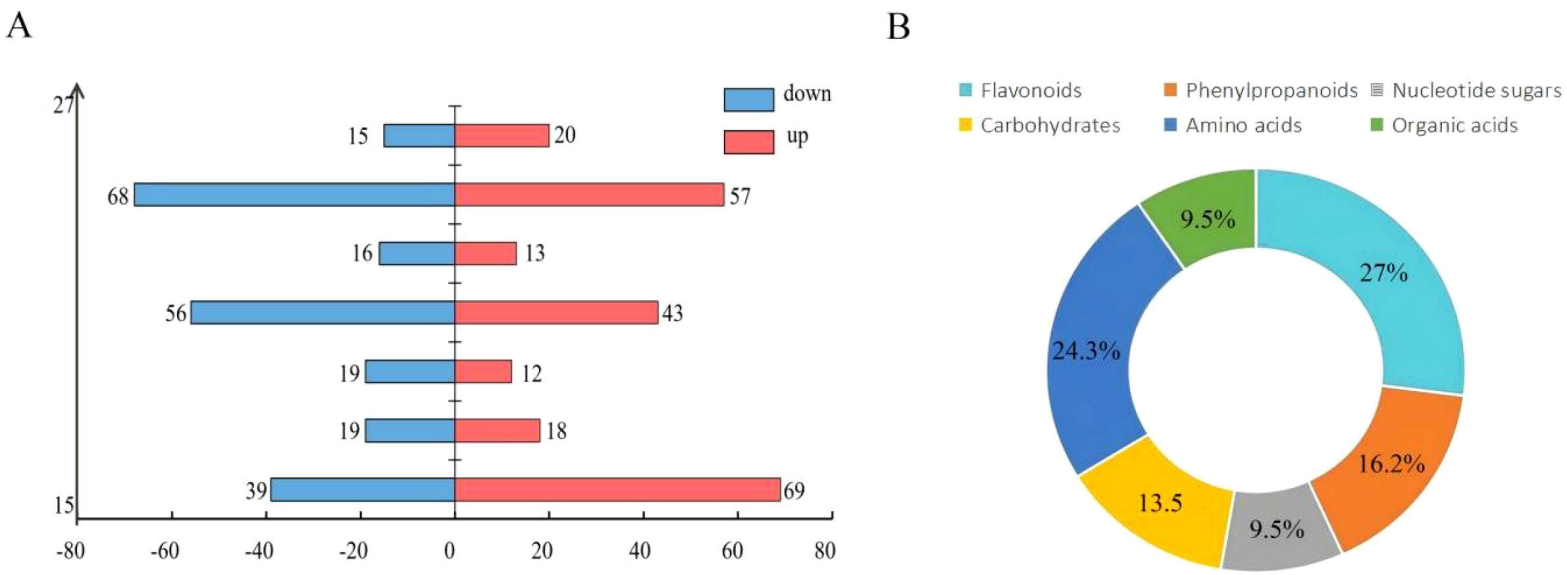
Metabolome sequencing results. **(A)** shows the number of differential metabolites at seven time points after Sr treatment(red indicating upregulated metabolites and blue indicating downregulated metabolites); **(B)** shows the proportion of different types of metabolites among anthocyanin-related metabolites.

To screen for biologically relevant differentially accumulated metabolites (DAMs), the following criteria were adopted: fold-change ≥ 1.2 or ≤ 0.83, and P-value < 0.05. This threshold was selected to sensitively detect typical moderate metabolic alterations in biological systems, resulting in the identification of 407 DAMs. These DAMs displayed distinct temporal distribution patterns across the seven time points: 15 days (108), 17 days (37), 19 days (31), 21 days (99), 23 days (29), 25 days (125), and 27 days (35). Notably, the highest number of DAMs (26.9%) was observed at 25 days, which exhibited a clear temporal association with the phase of sustained elevated strontium accumulation, suggesting that this stage may serve as a critical response node in the strontium-regulated metabolic network (**Figure 3A**). Subsequent pathway enrichment analysis and multi-omics integration further validated that the metabolite set screened using this threshold demonstrated high biological relevance.

From the total DAMs, 74 metabolites with direct or indirect links to the “phenylpropanoid-flavonoid” biosynthesis pathway were identified, constituting 15.9% of the total DAMs. These metabolites can be classified into six major functional categories: flavonoids, phenylpropanoids, nucleotide sugars, carbohydrates, amino acids, and organic acids (**Figure 3B**).

### 3.3 Screening of differential genes and correlation analysis with anthocyanin synthesis

**Figure 4:**
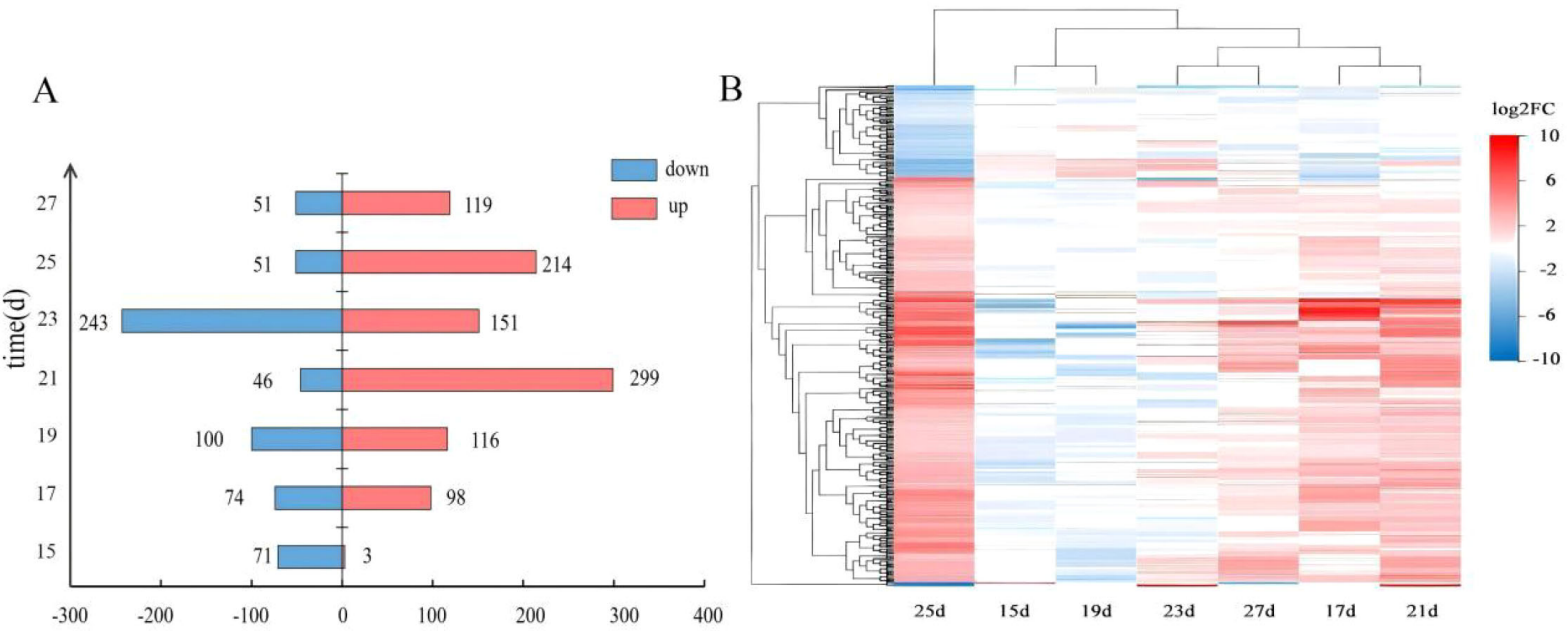
**(A)** displays the number of DEGs at each of the seven time points, with red and blue bars representing upregulated genes and downregulated genes, respectively. **(B)** presents a heatmap of DEG clustering, in which the vertical axis represents genes and the horizontal axis corresponds to the seven treatment time points. Reder colors indicate higher expression levels, while bluer colors denote lower expression levels.

High-throughput transcriptome sequencing of samples from seven developmental time points generated an average of 21,950,238 high-quality sequences. Data quality was robust, with the percentage of bases with quality scores (Phred) >30 consistently exceeding 91.07%. By comparing gene expression levels between the treatment and control groups (FDR Q-value < 0.05 and |Log2FC| ≥ 1), a total of 74, 172, 216, 345, 394, 265, and 170 DEGs were identified at 15, 17, 19, 21, 23, 25, and 27 DAP, respectively (**Figure 4A**). The quantity of DEGs reached a maximum at 21 and 23 DAP (345 and 394, respectively), directly coinciding with the peak of Sr content observed at 21 DAP (31.0247 mg/kg).

Heatmap clustering analysis (**Figure 4B**) revealed that DEG expression patterns segregated samples into two major modules. Samples from 15 and 19 DAP clustered together, while those from 21 and 23 DAP clustered together, indicating significant differences in the focus of transcriptomic responses regulated by Sr at different developmental stages. The global intersection analysis revealed no differentially expressed genes common to all seven time points, indicating that the transcriptional response induced by strontium treatment exhibits strong stage specificity.

GO enrichment analysis showed significant enrichment of DEGs in biological processes directly linked to anthocyanin synthesis at multiple time points. Significantly enriched terms included the phenylalanine biosynthetic process at 15 DAP (Q-value = 0.0449), the L-phenylalanine biosynthetic process at 17 DAP (Q-value = 0.0184), and the aromatic compound biosynthetic process at 25 DAP (Q-value = 0.054). Corresponding KEGG pathway enrichment analysis confirmed key structural genes in the phenylpropanoid and flavonoid biosynthesis pathways, such as *PAL* (Zhang and Liu, 2014), *DFR* (Jiang et al., 2023), and *C4H* (Jiang et al., 2024), exhibited significant upregulation (**Table 1**), aligning with the enhanced anthocyanin accumulation observed.

**Table 1:** Key rate-limiting genes enriched by KEGG.

ID	Gene name	KEGG pathway annotation	Log2FC	DAP
*Zm00001d003015*	*PAL*	L-phenylalanine ammonia-lyase (trans-cinnamate-forming)	2.750	25
*Zm00001d015513*	*DFR*	4-hydroxycinnamyl aldehyde:NADP+ oxidoreductase (CoA-hydroxycinnamoylating)	1.664	27
*Zm00001d016471*	*C4H*	trans-cinnamate,[reduced NADPH-–hemoprotein reductase]:oxygen oxidoreductase (4-hydroxylating)	3.210	27

### 3.4 Multi-omics integrated analysis of Sr-regulated anthocyanin synthesis

**Figure 5:**
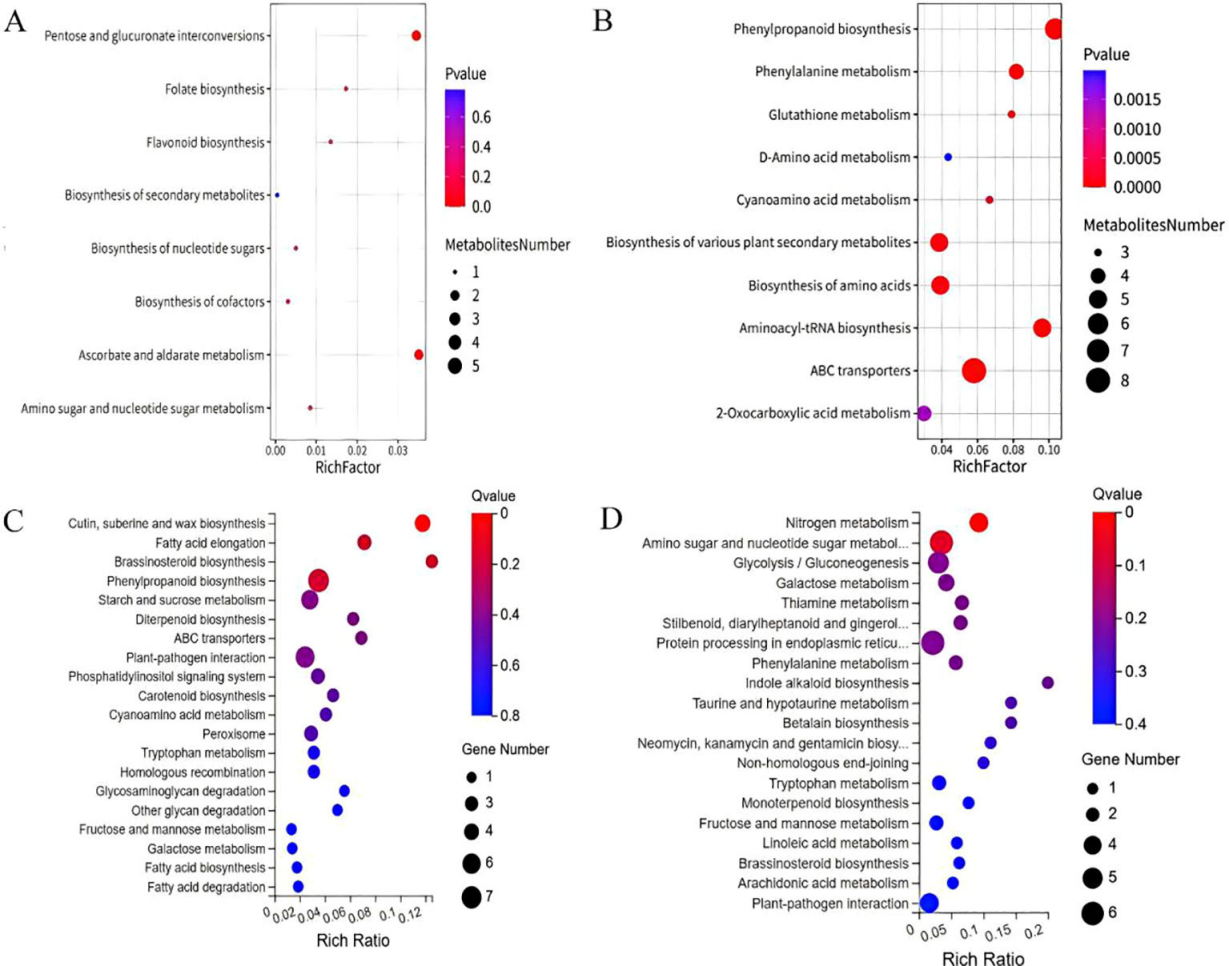
KEGG pathway enrichment bubble plots for metabolome and transcriptome at 23 and 25 days after treatment. **(A)** Metabolome at 23 days; **(B)** Metabolome at 25 days; **(C)** Transcriptome at 23 days; **(D)** Transcriptome at 25 days. Bubble size is the number of metabolites/genes enriched in the pathway; color indicates enrichment significance (-log_10_(Q value)).

KEGG pathway enrichment analysis across the 23 and 25 DAP time window revealed that Sr treatment induced a dramatic shift in the metabolic state within the purple maize kernels, correlating with the stage-specific pattern of anthocyanin accumulation. At 23 DAP, both metabolomic and transcriptomic data indicated significant activation of specific pathways, including flavonoid biosynthesis, amino sugar and nucleotide sugar metabolism, and phenylpropanoid biosynthesis. This concerted activation established the essential substrate and precursor foundation for subsequent anthocyanin production (**Figures 5A, B**). By 25 DAP, the enrichment pattern underwent a marked shift: at the metabolite level, glutathione metabolism and ABC transporter pathways were significantly enriched, indicating that cells had initiated antioxidant and stress response mechanisms; at the transcript level, enrichment shifted toward nitrogen metabolism, amino acid biosynthesis, glycolysis/gluconeogenesis, and stilbenoid biosynthesis pathways related to primary metabolism and defense responses (**Figures 5C, D**) (Waszczak et al., 2018). These data collectively suggest that following the peak of Sr accumulation, the metabolic focus shifts from secondary metabolite synthesis to a defense-adaptation phase centered on oxidative stress response and resource reallocation. This transitional reallocation may lead to the temporary diversion of some precursors originally intended for anthocyanin synthesis, explaining the slowdown in the rate of anthocyanin accumulation during this period (Huang et al., 2022). By 27 DAP, cells gradually established a new steady state, allowing metabolic resources to be reoptimized and ultimately driving a significant increase in anthocyanin content.

To elucidate the temporal association between Sr treatment and anthocyanin accumulation, Spearman correlation analysis was performed on transcriptomic and metabolomic data from the three key windows (23, 25, and 27 DAP). The analysis focused on genes with clear functional annotations and DAMs, enabling the construction of a time-resolved gene-metabolite co-correlation network. The analysis revealed that the Sr treatment response exhibits three dynamic stages, consistent with the “perception-defense-adaptation” metabolic reprogramming model common in plants responding to environmental stimuli (Mitra et al., 2021). At 23 DAP, the network was dominated by metabolites related to oxidized lipids and genes related to general stress response and cellular homeostasis, indicating an initial stress perception and adaptive adjustment phase triggered by Sr (Alarcón-Poblete et al., 2020). By 25 DAP, the network exhibited highly coordinated, functionally specific modules. Key phenylpropanoid/flavonoid derivatives showed significant positive correlations with gene clusters containing critical regulators (e.g., *C4H*, *MYB108-*like). Simultaneously, strong negative correlations were observed between these components and antioxidant molecules such as glutathione, clearly demonstrating a competitive trade-off for common precursors and reducing power between the phenylpropanoid/flavonoid synthesis pathway and the antioxidant system (Gentile et al., 2024; Khalid et al., 2024). This stage represents a key regulatory node for anthocyanin accumulation dynamics. By 27 DAP, the network converged on core modules linked to energy metabolism, developmental regulation, and redox homeostasis. This marks the transition to a metabolic optimization phase, where a newly established cellular steady state facilitates the efficient redirection of resources toward the final high-yield synthesis of anthocyanins (Ahmad et al., 2023).

### 3.5 qRT-PCR validation

To independently validate the transcriptome sequencing data at the experimental level and further delineate the temporal regulation of strontium treatment on anthocyanin synthesis-related gene expression, three core structural genes in the phenylpropane-flavonoid pathway, *PAL* (*Zm00001d003015*), *C4H* (*Zm00001d016471*), and *DFR* (*Zm00001d015513*), were selected for validation by qRT-PCR.

**Figure 6:**
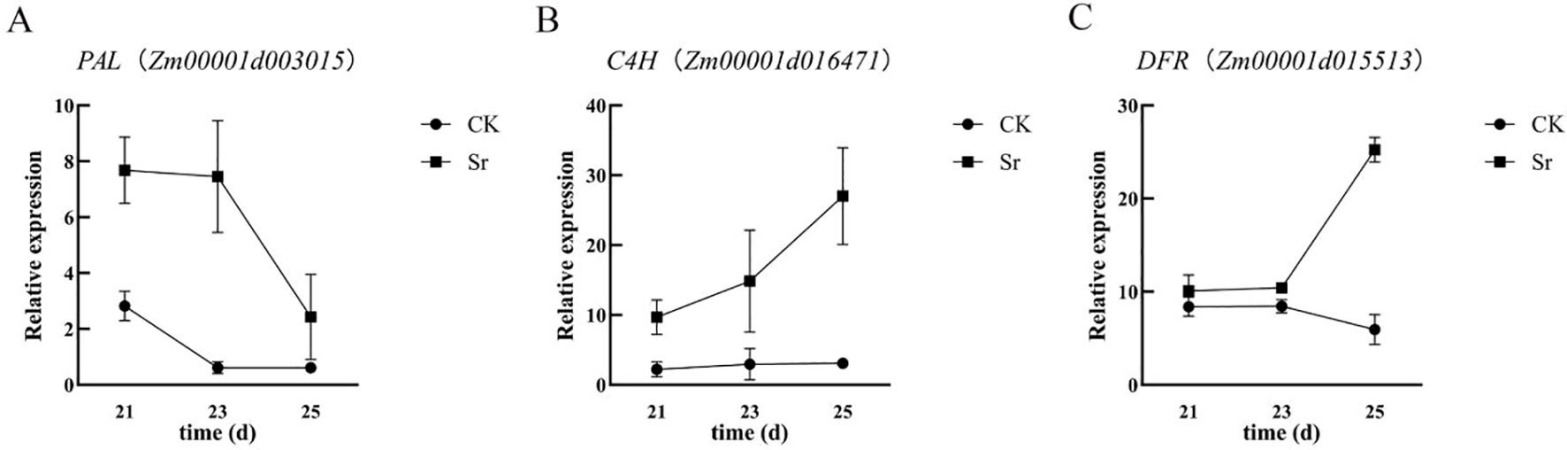
qRT-PCR results of key candidate genes. **(A-C)** respectively show the qRT-PCR results of the genes PAL, C4H, and DFR, where CK represents the gene expression level in the control group and Sr represents the gene expression level in the treated group.

The qRT-PCR results (**Figure 6**) demonstrated that the expression levels of all six candidate genes were significantly higher in the Sr treatment group (Sr) than those in the control group (CK) during their respective key developmental periods. Their temporal expression profiles were highly consistent with the trends identified in the transcriptome sequencing data. Specifically, the expression of *PAL*, *C4H*, and *DFR* showed an upward trend during the active phase of anthocyanin synthesis (23–27 DAP), which coincides with the period of upstream substrate consumption and downstream anthocyanin precursor accumulation detected in the metabolome. These findings further support the conclusion that Sr treatment transcriptionally enhances the flux through the phenylpropanoid-flavonoid pathway.

## 4 Discussion

### 4.1 Sr treatment as an upstream signal inducing anthocyanin accumulation and triggering systemic reprogramming

The present study demonstrates that exogenous application of Sr at optimal concentrations serves as a potent inducer, significantly enhancing anthocyanin biosynthesis in fresh purple maize kernels (**Figure 2B**). This physiological response is consistent with the well-established observation that various trace elements can stimulate plant secondary metabolism (Bhat et al., 2020; Akhtar et al., 2026). Notably, longitudinal monitoring across multiple time points revealed a definitive causal temporal sequence between Sr accumulation and anthocyanin synthesis. As illustrated in **Figure 2A**, Sr content reached its peak at 21 DAP (31.0247 mg/kg), whereas the most rapid phase of anthocyanin accumulation occurred subsequently, between 23 and 27 DAP (**Figure 2B**). This distinct temporal lag identifies Sr accumulation as a pivotal upstream event that initiates the subsequent enhancement of anthocyanin biosynthesis, conforming to the canonical “perception-transduction-response” model of plant signaling (Xiao-yan et al., 2023; Sun et al, 2013). Furthermore, these results provide empirical evidence elucidating the “low-concentration promotion and high-concentration inhibition” effect of trace elements in plants (Greger et al., 2018; dos Santos et al., 2024).

Transcriptomic profiling offers molecular insights into this signal transduction process. The number of differentially expressed genes (DEGs) peaked at 21 and 23 DAP (345 and 394, respectively) (**Figure 4A**), immediately following the peak in Sr accumulation. The absence of universally shared DEGs across all seven time points suggests that Sr triggers stage-specific transcriptional reprogramming, activating distinct gene modules within different developmental windows. Furthermore, multi-omics principal component analysis corroborated that Sr treatment systematically and directionally modulated the metabolic landscape of the kernels at both the transcriptomic and metabolomic levels during the critical period of anthocyanin synthesis (23–27 DAP).

### 4.2 Temporally coordinated activation of the phenylpropanoid-flavonoid pathway is the core molecular mechanism

Integrated multi-omics analyses indicate that Sr enhances anthocyanin biosynthesis predominantly via the coordinated temporal activation of the phenylpropanoid-flavonoid pathway, as evidenced by the robust coupling between transcriptional shifts and metabolic fluctuations.

At the transcriptional level, key rate-limiting enzymes were specifically upregulated during the active phase of anthocyanin accumulation. As detailed in **Table 1**, phenylalanine ammonia-lyase (*PAL*, *Zm00001d003015*) was significantly upregulated at 25 DAP (Log2FC = 2.750), while cinnamate 4-hydroxylase (*C4H*, *Zm00001d016471*) and dihydroflavonol 4-reductase (*DFR*, *Zm00001d015513*) exhibited upregulation at 27 DAP (Log2FC = 3.210 and 1.664, respectively). This sequential activation pattern represents the primary transcriptional framework driving the pathway (Chen et al., 2022; Aravena-Calvo et al., 2024; Zhang et al., 2025).

Metabolically, the dynamics of central hub metabolites reflect the functional consequences of this transcriptional activation. According to metabolomic data (**Supplementary Table 1**), core phenylpropanoid intermediates, including 4-coumaric acid (Log2FC = 7.54) and 3-coumaric acid (Log2FC = 6.86), accumulated dramatically during the critical 25 DAP stage. Simultaneously, the upstream substrates L-phenylalanine and L-tyrosine were significantly depleted (Log2FC = -2.07 and -1.84, respectively), while downstream flavonoid derivatives, such as polydatin, showed massive accumulation at 21 and 25 DAP (Log2FC = 10.19 and 11.48). This “precursor depletion–intermediate/product accumulation” pattern provides direct evidence for the redirection of metabolic flux into the phenylpropanoid-flavonoid pathway (Sanchez-Henao et al., 2021; Yuan et al., 2016). Such synergistic patterns are characteristic of metabolic flux redirection toward specific secondary metabolic pathways (Wang et al., 2022).

KEGG enrichment analysis bridges these transcriptional and metabolic shifts. At 23 DAP, both datasets showed significant activation of “Flavonoid biosynthesis,” “Phenylpropanoid biosynthesis,” and “Amino sugar and nucleotide sugar metabolism” pathways (**Figures 5A, C**). This suggests that during the initiation phase of anthocyanin synthesis, the core biosynthetic pathway was upregulated in tandem with auxiliary pathways responsible for providing glycosylation substrates (e.g., UDP-sugars) (Tzin and Galili, 2010; Yoo et al., 2013; Ozeki et al., 2020). Together, these form an efficient and integrated “precursor supply–core synthesis–product modification” metabolic module (Jariani et al., 2024; Tullus et al., 2021).

### 4.3 Dynamic remodeling of metabolic networks and resource competition shape the dynamic process of synthesis

The metabolic response to Sr treatment was non-linear, characterized by a dynamic interplay of competition, trade-offs, and homeostatic re-balancing, which collectively shaped the phased dynamics of anthocyanin accumulation.

Combined pathway enrichment analysis highlighted this dynamic progression. Following the peak in Sr accumulation, the enrichment patterns shifted markedly at 25 DAP (**Figures 5B, D**). At the metabolite level, pathways such as “Glutathione metabolism” were enriched, suggesting the induction of antioxidant defense and stress-responsive systems. Concurrently, at the transcript level, enrichment shifted toward “Nitrogen metabolism,” “Amino acid biosynthesis,” and “Glycolysis/Gluconeogenesis.” This transition implies that post-peak Sr levels initiated a comprehensive stress adaptation program, potentially leading to competition for metabolic resources (e.g., phenylalanine and reducing power) between the phenylpropanoid/flavonoid pathway and antioxidant defense systems (Zhang et al., 2022, 2023). Such internal competition likely underlies the phased fluctuations observed in the rate of anthocyanin accumulation.

**Figure 7:**
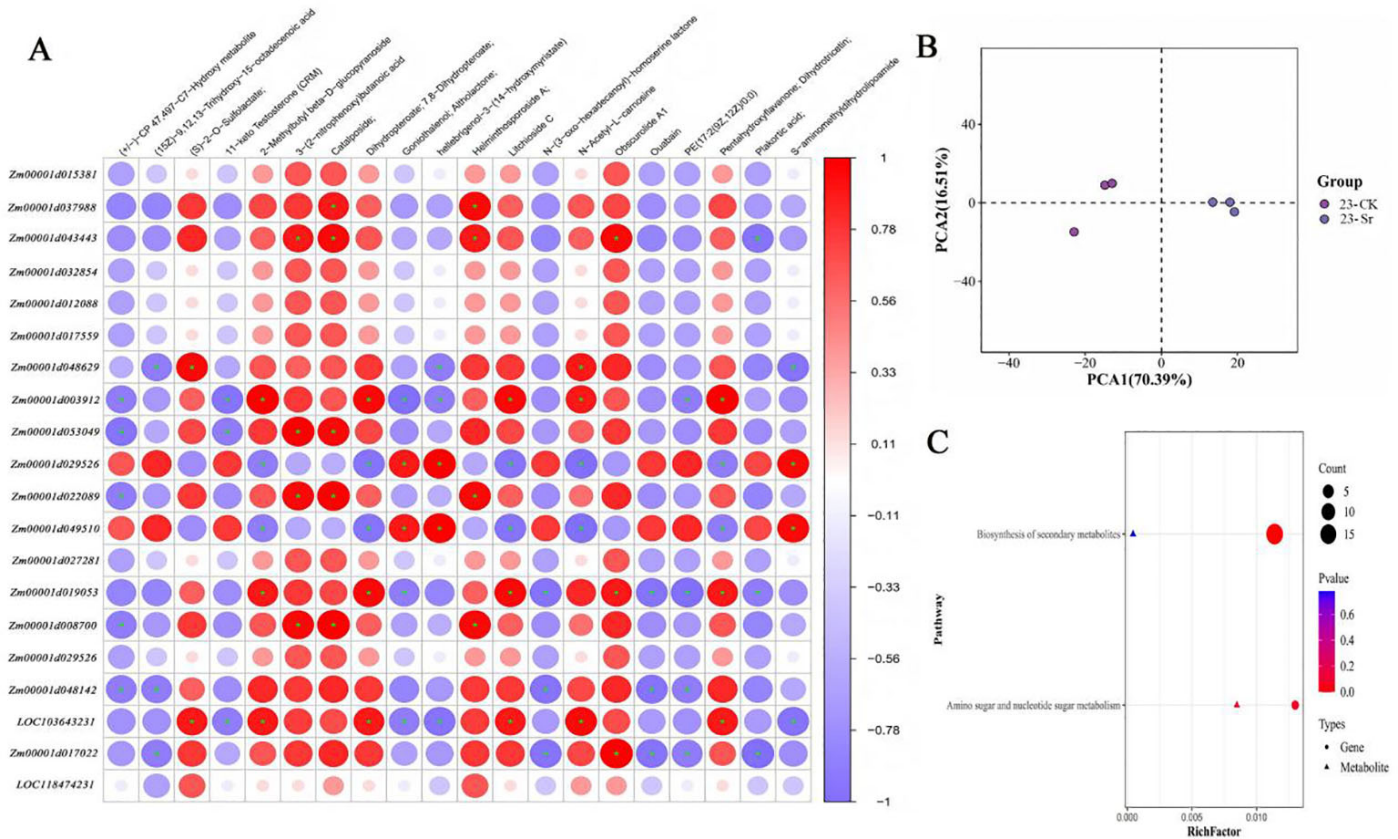
23 d combined analysis results. **(A)** shows the Spearman correlation analysis, where each column represents a differential metabolite and each row represents a differential indicator associated with the omics. The matrix color blocks indicate the Spearman rank correlation coefficient, with darker colors representing stronger correlations between differential metabolites and the associated omics indicators. Red indicates positive correlation, while blue indicates negative correlation. Symbols * denote p < 0.05, and ** denote p < 0.01. **(B)** presents the metabolic and associated omics PCA plot. **(C)** is a pathway enrichment analysis bubble plot, with the X-axis showing the enrichment factor (RichFactor). A higher value indicates a greater proportion of differential metabolites annotated to the pathway compared to the associated omics indicators. Circular shapes in the figure represent the indicator pathways of the associated omics, triangles represent metabolic pathways, the size of the shapes indicates the number of differential metabolites annotated to the pathway relative to the associated omics indicators, the larger the circle or triangle, the more indicators it represents, and the color of the shapes signifies pathway significance.

Multi-omics correlation analysis provided further substantiation (**Figure 7A**). During key regulatory windows (e.g., 25 DAP), phenylpropanoid/flavonoid metabolites exhibited significant positive correlations with biosynthetic genes like C4H, but negative correlations with specific antioxidant-related molecules, illustrating the resource allocation trade-offs between diverging metabolic branches. Additionally, as suggested in the Abstract, Sr treatment may optimize resource partitioning by downregulating competing metabolic branches (e.g., coumarin biosynthesis) (Tullus et al., 2021; Lv et al., 2024), thereby funneling precursors such as coumaroyl-CoA specifically toward anthocyanin biosynthesis.

By the late developmental stage (27 DAP), the gene-metabolite association network transitioned toward pathways involved in energy metabolism and redox homeostasis (**Supplementary Figure 2**). This indicates that the cells established a novel homeostatic state following the Sr-induced response and subsequent metabolic competition. In this state, the consumption of primary carbon sources and enhanced glycosylation capacity could be effectively harnessed to support the robust final-stage accumulation and stabilization of anthocyanins.

## 5 Conclusion

This study systematically elucidated the physiological effects and molecular regulatory mechanisms by which exogenous Sr treatment promotes anthocyanin synthesis in fresh purple maize via a temporally coordinated regulatory network. The results demonstrated that Sr treatment not only significantly enhanced the accumulation of Sr and anthocyanins in kernels but also showed a significant positive correlation between the two. Integrated multi-omics analysis further delineated a multi−layered regulatory network driven by Sr. Integrated multi-omics analysis further revealed that strontium treatment redirects the directional shift of metabolic flux toward the anthocyanin synthesis pathway by upregulating the expression of key rate-limiting enzymes such as *PAL*, *C4H*, and *DFR* in the phenylpropanoid-flavonoid pathway. This shift is coordinated with alterations in glycosylation-related pathways, forming a multi-layered regulatory network that integrates metabolites, pathways, and genes. By focusing on the core pathway of anthocyanin synthesis, this study systematically elucidates the promoting mechanism of strontium and provides a theoretical foundation along with key candidate genes for developing strontium-enriched, high-anthocyanin maize. Future research should explore the specific perception and transduction mechanisms of strontium signaling, functionally validate key genes, and evaluate the stability and applicability of this regulatory network under field production conditions.

## Data Availability

The datasets presented in this study can be found in online repositories. The names of the repository/repositories and accession number(s) can be found in the article/**Supplementary Material**.

## References

[B1] Ahmad I. Zhu G. Zhou G. Liu J. Younas M. U. Zhu Y. (2023). Melatonin role in plant growth and physiology under abiotic stress. Int. J. Mol. Sci. 24, 8759. doi: 10.3390/ijms24108759, PMID: 37240106 PMC10218329

[B2] Akhtar M. T. Lu Z. Ren S. Zou H. Noor I. Jin B. (2026). Copper homeostasis: Crosstalk with plant secondary metabolism and stress responses. Plant Sci. 362, 112795. doi: 10.1016/j.plantsci.2025.112795, PMID: 41038581

[B3] Alarcón-Poblete E. González-Villagra J. de Oliveira Silva F. M. Nunes-Nesi A. Inostroza-Blancheteau C. Alberdi M. . (2020). Metabolic responses of Vaccinium corymbosum L. cultivars to Al3+ toxicity and gypsum amendment. Environ. Exp. Bot. 176, 104119. doi: 10.1016/j.envexpbot.2020.104119, PMID: 41792008

[B4] Aravena-Calvo J. Busck-Mellor S. Laursen T. (2024). Global organization of phenylpropanoid and anthocyanin pathways revealed by proximity labeling of trans-cinnamic acid 4-hydroxylase in Petunia inflata petal protoplasts. Front. Plant Sci. 15. doi: 10.3389/fpls.2024.1295750, PMID: 39363925 PMC11446795

[B5] Bhat B. A. Islam S. T. Ali A. Sheikh B. A. Tariq L. Islam S. U. . (2020). Role of micronutrients in secondary metabolism of plants. In Plant Micronutrients pp, 311–329. doi: 10.1007/978-3-030-49856-6_13, PMID: 41790318

[B6] Borciani G. Ciapetti G. Vitale-Brovarone C. Baldini N. (2022). Strontium functionalization of biomaterials for bone tissue engineering purposes: A biological point of view. Materials 15, 1724. doi: 10.3390/ma15051724, PMID: 35268956 PMC8911212

[B7] Cappellini F. Marinelli A. Toccaceli M. Tonelli C. Petroni K. (2021). Anthocyanins: from mechanisms of regulation in plants to health benefits in foods. Front. Plant Sci. 12. doi: 10.3389/fpls.2021.748049, PMID: 34777426 PMC8580863

[B8] Chang S. Wang J. Wang J. (2017). Effects of strontium stress on growth and physiological/biochemical responses of maize seedlings. Hubei Agric. Sci. 56, 32–34 + 38. doi: 10.14088/j.cnki.issn0439-8114.2017.01.009

[B9] Chao E. Wu M. Yue D. Yuan Y. Qiu N. Zhou F. (2024). Promoting effect of low concentration strontium on photosynthetic performance of Chinese cabbage seedlings: Combined leaf characteristics, photosynthetic carbon assimilation and chlorophyll fluorescence. Ecotoxicol. Environ. Saf. 274, 116200. doi: 10.1016/j.ecoenv.2024.116200, PMID: 38479316

[B10] Chen X. Wang P. Gu M. Hou B. Zhang C. Zheng Y. . (2022). Identification of *PAL* genes related to anthocyanin synthesis in tea plants and its correlation with anthocyanin content. Hortic. Plant J. 8, 381–394. doi: 10.1016/j.hpj.2021.12.005, PMID: 41792008

[B11] Cheng X. Chen C. Hu Y. Guo X. Wang J. (2022). Photosynthesis and growth of Amaranthus tricolor under strontium stress. Chemosphere 308, 136234. doi: 10.1016/j.chemosphere.2022.136234, PMID: 36041533

[B12] Cock P. J. A. Fields C. J. Goto N. Heuer M. L. Rice P. M. (2009). The Sanger FASTQ file format for sequences with quality scores, and the Solexa/Illumina FASTQ variants. Nucleic Acids Res. 38, 1767–1771. doi: 10.1093/nar/gkp1137, PMID: 20015970 PMC2847217

[B13] dos Santos L. C. Martins G. S. de Sousa Lima J. da Silva G. A. M. Nunes M. F. P. N. de Oliveira I. P. . (2024). Enhancing wheat resilience to water deficit through selenium biofortification: perspectives on physiological, biochemical and nutritional responses. J. Soil Sci. Plant Nutr. 24, 7418–7435. doi: 10.1007/s42729-024-02049-5, PMID: 41792474

[B14] Gao F. Yang P. Wang W. Wang K. Zhao L. Wang Y. . (2025). Unveiling the multifaceted roles of anthocyanins: a review of their bioavailability, impacts on gut and system health, and industrial implications. Curr. Res. Food Sci. 11, 101137. doi: 10.1016/j.crfs.2025.101137, PMID: 40703150 PMC12284573

[B15] Gentile D. Serino G. Frugis G. (2024). CRF transcription factors in the trade-off between abiotic stress response and plant developmental processes. Front. Genet. 15. doi: 10.3389/fgene.2024.1377204, PMID: 38694876 PMC11062136

[B16] Greger M. Landberg T. Vaculík M. (2018). Silicon influences soil availability and accumulation of mineral nutrients in various plant species. Plants 7, 41. doi: 10.3390/plants7020041, PMID: 29783754 PMC6027514

[B17] Hong T. Zhao Z. Bian W. Zhu W. Li Z. Shen G. . (2023). Development of a novel nutritional assessment model based on strontium and other compositional factors in apples across seven regions in China. Front. Sustain. Food Syst. 7. doi: 10.3389/fsufs.2023.1292999, PMID: 41789077

[B18] Huang W. Han S. Wang L. Li W. (2022). Carbon and nitrogen metabolic regulation in freshwater plant Ottelia alismoides in response to carbon limitation: A metabolite perspective. Front. Plant Sci. 13. doi: 10.3389/fpls.2022.962622, PMID: 36186073 PMC9522611

[B19] Jariani P. Shahnejat-Bushehri A.-A. Naderi R. Zargar M. Naghavi M. R. (2024). Characterization of key genes in anthocyanin and flavonoid biosynthesis during floral development in Rosa canina L. Int. J. Biol. Macromolecules 276, 133937. doi: 10.1016/j.ijbiomac.2024.133937, PMID: 39029843

[B20] Jiang L. Yang Y. Zhou Z. Chen X. (2024). Physiological Studies and Transcriptomic Analysis Reveal the Mechanism of Saline-Alkali Stress Resistance of Malus sieversii f. niedzwetzkyan. Horticulturae 10, 510. doi: 10.3390/horticulturae10050510, PMID: 41725453

[B21] Jiang L. Yue M. Liu Y. Zhang N. Lin Y. Zhang Y. . (2023). A novel &60;scp&62;R2R3-MYB&60;/scp&62; transcription factor &60;scp&62;FaMYB5&60;/scp&62; positively regulates anthocyanin and proanthocyanidin biosynthesis in cultivated strawberries (Fragaria × ananassa). Plant Biotechnol. J. 21, 1140–1158. doi: 10.1111/pbi.14024, PMID: 36752420 PMC10214752

[B22] Khalid F. Rasheed Y. Asif K. Ashraf H. Maqsood M. F. Shahbaz M. . (2024). Plant biostimulants: mechanisms and applications for enhancing plant resilience to abiotic stresses. J. Soil Sci. Plant Nutr. 24, 6641–6690. doi: 10.1007/s42729-024-01996-3, PMID: 41792474

[B23] Lao F. Sigurdson G. T. Giusti M. M. (2017). Health benefits of purple corn (Zea mays L.) phenolic compounds. Compr. Rev. Food Sci. Food Saf. 16, 234–246. doi: 10.1111/1541-4337.12249, PMID: 33371534

[B24] Li F. Sun Q. Chen L. Zhang R. Zhang Z. (2024). Unlocking the health potential of anthocyanins: a structural insight into their varied biological effects. Crit. Rev. Food Sci. Nutr. 65, 2134–2154. doi: 10.1080/10408398.2024.2328176, PMID: 38494796

[B25] Lv J. Yang S. Zhou W. Liu Z. Tan J. Wei M. (2024). Microbial regulation of plant secondary metabolites: Impact, mechanisms and prospects. Microbiological Res. 283, 127688. doi: 10.1016/j.micres.2024.127688, PMID: 38479233

[B26] Mitra A. Kataki S. Singh A. N. Gaur A. Razafindrabe B. H. N. Kumar P. . (2021). “ Plant Stress, Acclimation, and Adaptation: A Review,” in Plant in Challenging Environments ( Springer International Publishing), 1–22. doi: 10.1007/978-3-030-78420-1_1, PMID:

[B27] Ozeki Y. Iijima L. Higuchi K. Miyahara T. Sasaki N. Tsujimoto T. . (2020). “ Molecular mechanisms of carnation flower colors via anthocyanin and flavonoid biosynthetic pathways,” in Compendium of Plant Genomes (Singapore: Springer), 99–117. doi: 10.1007/978-981-15-8261-5_8, PMID:

[B28] Ramadani M. R. N. Jadid N. (2024). A comprehensive review of *in vitro* precursor feeding strategies for the overproduction of high-value plant secondary metabolites. Arabian J. Chem. 17, 106018. doi: 10.1016/j.arabjc.2024.106018, PMID: 41792008

[B29] Ru X. Yang L. Shen G. Wang K. Xu Z. Bian W. . (2024). Microelement strontium and human health: comprehensive analysis of the role in inflammation and non-communicable diseases (NCDs). Front. Chem. 12. doi: 10.3389/fchem.2024.1367395, PMID: 38606081 PMC11007224

[B30] Sahin O. Babar S. K. Deniz K. Kadioglu Y. K. Gunes A. (2024). Strontium biofortification in soil and hydroponic grown tomato and lettuce. J. Plant Nutr. 47, 1456–1463. doi: 10.1080/01904167.2024.2315984, PMID: 41783271

[B31] Sanchez-Henao C. P. Ramirez-Malule H. Lopez-Agudelo V. A. (2021). “ Basic concepts of metabolic flux analysis,” ( INGENIERIA Y COMPETITIVIDAD).

[B32] Sasmaz M. Uslu Senel G. Obek E. (2020). Strontium accumulation by the terrestrial and aquatic plants affected by mining and municipal wastewaters (Elazig, Turkey). Environ. Geochem. Health 43, 2257–2270. doi: 10.1007/s10653-020-00629-9, PMID: 32728950

[B33] Sun X.-L. Yu Q.-Y. Tang L.-L. Ji W. Bai X. Cai H. . (2013). GsSRK, a G-type lectin S-receptor-like serine/threonine protein kinase, is a positive regulator of plant tolerance to salt stress. J. Plant Physiol. 170, 505–515. doi: 10.1016/j.jplph.2012.11.017, PMID: 23276523

[B34] Tullus A. Rusalepp L. Lutter R. Rosenvald K. Kaasik A. Rytter L. . (2021). Climate and competitive status modulate the variation in secondary metabolites more in leaves than in fine roots of betula pendula. Front. Plant Sci. 12. doi: 10.3389/fpls.2021.746165, PMID: 34899775 PMC8655902

[B35] Tzin V. Galili G. (2010). The Biosynthetic Pathways for Shikimate and Aromatic Amino Acids inArabidopsis thaliana. Arabidopsis Book 8, e0132. doi: 10.1199/tab.0132, PMID: 22303258 PMC3244902

[B36] Wang S. Li Y. He L. Yang J. Fernie A. R. Luo J. (2022). Natural variance at the interface of plant primary and specialized metabolism. Curr. Opin. Plant Biol. 67, 102201. doi: 10.1016/j.pbi.2022.102201, PMID: 35349968

[B37] Waszczak C. Carmody M. Kangasjärvi J. (2018). Reactive oxygen species in plant signaling. Annu. Rev. Plant Biol. 69, 209–236. doi: 10.1146/annurev-arplant-042817-040322, PMID: 29489394

[B38] Xiao-yan Z. Shu-hua Y. Yang-lin D. (2023). Molecular Mechanism of Cold Signal Perception and Transduction in Plants ( Biotechnology Bulletin).

[B39] Yoo H. Widhalm J. R. Qian Y. Maeda H. Cooper B. R. Jannasch A. S. . (2013). An alternative pathway contributes to phenylalanine biosynthesis in plants via a cytosolic tyrosine:phenylpyruvate aminotransferase. Nat. Commun. 4. doi: 10.1038/ncomms3833, PMID: 24270997

[B40] Yuan H. Cheung C. Y. M. Hilbers P. A. J. van Riel N. A. W. (2016). Flux balance analysis of plant metabolism: the effect of biomass composition and model structure on model predictions. Front. Plant Sci. 7. doi: 10.3389/fpls.2016.00537, PMID: 27200014 PMC4845513

[B41] Zhang Y. Guo S. Zhang Z. Li R. Du S. Hao S. . (2025). Functional characterization of anthocyanin biosynthesis-related dihydroflavonol 4-reductase (*DFR*) genes in blueberries (Vaccinium corymbosum). Plants 14, 1449. doi: 10.3390/plantSr4101449, PMID: 40431014 PMC12114909

[B42] Zhang W. Kang Z. Wang Q. Qiu N. Chen M. Zhou F. (2020). The biological effects of strontium (^88^Sr) on Chinese cabbage. Plant Soil Environ. 66, 149–154. doi: 10.17221/108/2020-pse

[B43] Zhang X. Liu C.-J. (2014). Multifaceted regulations of gateway enzyme phenylalanine ammonia-lyase in the biosynthesis of phenylpropanoids. Mol. Plant. doi: 10.1093/mp/ssu134, PMID: 25385698

[B44] Zhang D. Liu J. Zhang Y. Wang H. Wei S. Zhang X. . (2023). Morphophysiological, proteomic and metabolomic analyses reveal cadmium tolerance mechanism in common wheat (Triticum aestivum L.). J. Hazardous Materials 445, 130499. doi: 10.1016/j.jhazmat.2022.130499, PMID: 36455318

[B45] Zhang L. Zhang L. Kang L. (2022). Promoter cloning of PuLOX2S gene from “Nanguo” pears and screening of transcription factors by Y1H technique. J. Food Biochem. 46. doi: 10.1111/jfbc.14278, PMID: 35748399

