# Supplementary material for: Multi-omics analysis of the metabolic and transcriptional regulatory mechanisms underlying strontium-induced anthocyanin accumulation in fresh purple maize
Source: Front Plant Sci. 2026 Mar 12;17:1777794. doi: 10.3389/fpls.2026.1777794 (PMC13019636; doi:10.3389/fpls.2026.1777794)
Supplement: Supplementary file 9 [file Table9.docx]

Addendum

Table 1 Anthocyanin-related differential metabolites

| Metabolite Name | Time（DAP） | Trend | Log2 |
| --- | --- | --- | --- |
| Flavonoids | | | |
| Eupatilin | 15 | Up | 3.12 |
| Hesperetin | 17 | Down | 2.86 |
| Quercetin 3-(2G-xylosylrutinoside) | 21 | Down | 3.32 |
| Naringin chalcone | 19 | Up | 1.69 |
| Kaempferol 3-O-beta-D-xyloside | 21 | Down | 3.61 |
| Polydatin | 21 | Up | 10.19 |
| Homoplantaginin | 21 | Up | 1.58 |
| Luteolin 7-rutinoside-3'-glucoside | 21 | Up | 2.07 |
| Hesperetin 7-O-glucoside | 21 | Up | 4.31 |
| Luteolin 3',4'-diglucuronide | 23 | Down | 8.53 |
| Pentahydroxyflavanone | 23 | Down | 2.29 |
| Lysionotin | 25 | Up | 6.57 |
| Polydatin | 25 | Up | 11.48 |
| Neodiosmin | 25 | Down | 1.31 |
| Gossypin | 25 | Down | 4.2 |
| Pachyrrhizone | 25 | Down | 3.18 |
| Isophylloflavanine | 27 | Down | 2.98 |
| 5,7-Dihydroxychromone | 17 | Down | 3.29 |
| 5,2'-Dihydroxy-6,7,8,6'-tetramethoxyflavone | 17 | Up | 4.9 |
| Epigallocatechin 3-O-caffeate | 19 | Up | 1.36 |
| Phenylpropanoids | | | |
| 3,5-Dimethoxy-4-hydroxybenzaldehyde | 15 | Up | 2.81 |
| Ethyl gallate | 19 | Up | 2.24 |
| 3,5-Dimethoxy-4-hydroxybenzaldehyde | 21 | Up | 2.28 |
| 4-Hydroxybenzoic acid | 21 | Down | 1.4 |
| 3-Coumaric acid | 25 | Up | 6.86 |
| 4-Coumaric acid | 25 | Up | 7.54 |
| 3,5-Dimethoxy-4-hydroxybenzaldehyde | 25 | Up | 4.42 |
| 2-Hydroxycinnamic acid | 25 | Down | 2.17 |
| Gallic acid trimethyl ether | 27 | Up | 1.84 |
| Gentisic acid | 15 | Up | 2.41 |
| 5-Hydroxyferulic acid methyl ester; | 19 | Down | 1.85 |
| Nucleotide sugars | | | |
| UDP; Uridine 5'-diphosphate | 19 | Up | 12.04 |
| Swertianolin | 21 | Down | 1.37 |
| 2-Methylbutyl beta-D-glucopyranoside | 23 | Down | 3.14 |
| (-)-Syringaresinol di-O-glucoside | 25 | Up | 20.36 |
| 1-Caffeoyl-beta-D-glucose | 25 | Up | 3.03 |
| [5-(2-thienyl)-3-isoxazolyl] methanol | 25 | Up | 1.78 |
| Carbohydrates | | | |
| D-(-)-Arabinose | 15 | Up | 1.83 |
| Raffinose | 17 | Up | 1.65 |
| Lactobionic acid | 19 | Up | 1.57 |
| D-Raffinose | 21 | Down | 1.51 |
| D-(-)-Arabinose | 23 | Down | 1.75 |
| 1-Caffeoyl-beta-D-glucose | 25 | Up | 3.03 |
| alpha-Lactose | 25 | Down | 4.31 |
| Sucrose | 25 | Down | 4.28 |
| Volemitol; D-glycero-D-manno-Heptitol | 27 | Up | 11.15 |
| D-Glucose | 19 | Down | 1.39 |
| Amino acids | | | |
| Leucylproline | 19 | Up | 1.51 |
| N-Acetyl-L-carnosine | 23 | Down | 4.51 |
| Spermidine | 25 | Down | 3.86 |
| L-Glutamic acid | 25 | Down | 2.28 |
| L-Tyrosine | 25 | Down | 1.84 |
| L-Phenylalanine | 25 | Down | 2.07 |
| L-Isoleucine | 25 | Down | 2.39 |
| N-stearoyl glutamic acid | 25 | Down | 1.81 |
| N-benzyl-2-(6-methoxy-2-naphthyl) propanamide | 15 | Up | 1.45 |
| 6-Methylindole | 15 | Up | 1.38 |
| N-Acetyl-D-mannosaminolactone | 19 | Up | 1.62 |
| Pantetheine | 19 | Up | 1.49 |
| 3-Hydroxyanthranilate | 19 | Up | 1.53 |
| L-(+)-Arginine | 27 | Up | 1.66 |
| N-palmitoyl methionine | 19 | Down | 1.42 |
| N,N-Dihydroxy-L-tyrosine | 21 | Down | 2.46 |
| Organic acids | | | |
| Gluconic acid | 15 | Up | 1.75 |
| Methyl acetoacetate | 17 | Down | 1.44 |
| 4-acetyl-4-(ethoxycarbonyl) heptanedioic acid | 17 | Up | 1.48 |
| Methyl acetoacetate | 21 | Down | 1.63 |
| Azelaic acid | 21 | Down | 1.58 |
| Trimellitic anhydride | 25 | Up | 5.22 |
| delta-Gluconic acid delta-lactone | 27 | Down | 1.37 |

Table 2 Internal reference genes and primer sequences

| ID | sequence |
| --- | --- |
| qZmActin2-F | TACGCTAGTGGGCGAACAAC |
| qZmActin2-R | CATTAGGTGGTCGGTGAGGT |
| GAPDH-F | ACTTCGGCATTGTTGAGG |
| GAPDH-R | AAGTCGGTAGAAACCAGAT |
| C4H-F | ACCCCGGAGAACGTCATCTA |
| C4H-R | GATGGTGGACTCCGTGATGG |
| CCR1-F | AAGCGAACCGACAACCTGTA |
| CCR1-R | ACGTGGTTGGTGCTGAAGTT |
| PAL-F | ATGGAGTGCGAGAACGGACAC |
| PAL-R | CTTCACGGCATCAAGGTGGC |
| DFR-F | AAGAGCACCGAGAACTGGTA |
| DFR-R | CTTTGTCTGTCCCGTGAGGT |
| CAD-F | GAAGGCAGCACCTCTTCTGT |
| CAD-R | CATGTGACCCAGACCACCAA |


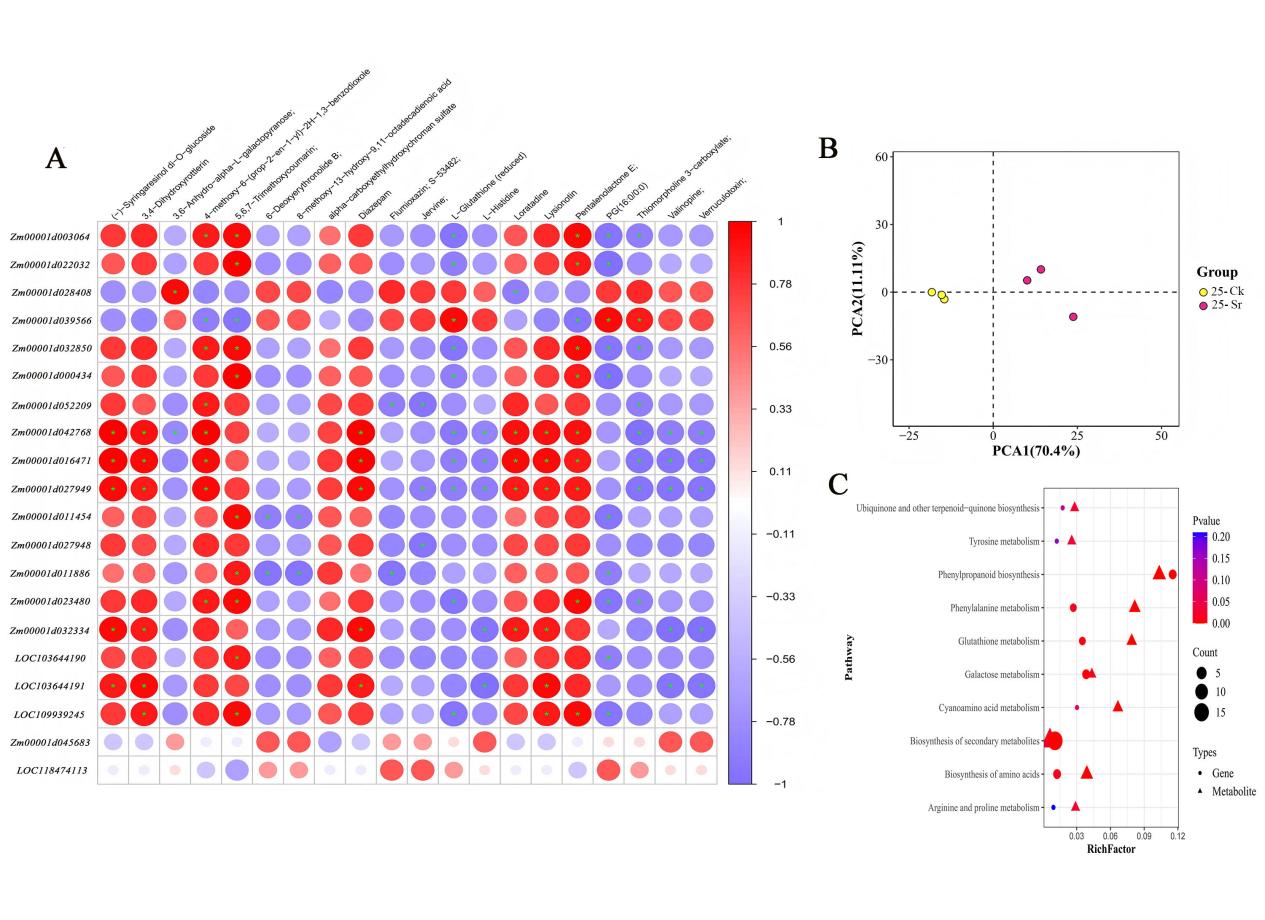


Figure 1 Combined analysis results of 25 DAP


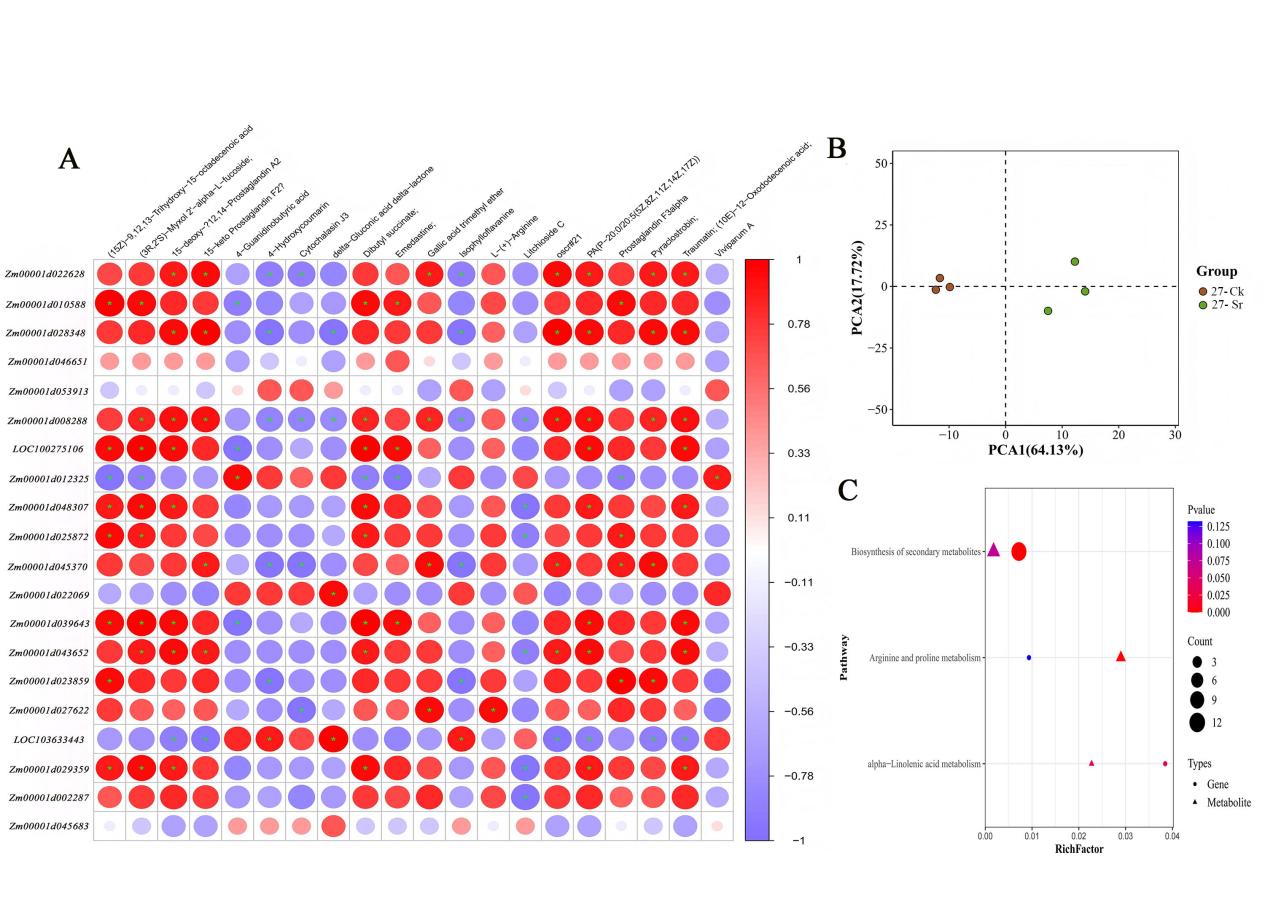


Figure 2 27d Combined analysis results
